# Endocrinology of the Aging Prostate: Current Concepts

**DOI:** 10.3389/fendo.2021.554078

**Published:** 2021-02-22

**Authors:** Rossella Cannarella, Rosita A. Condorelli, Federica Barbagallo, Sandro La Vignera, Aldo E. Calogero

**Affiliations:** Department of Clinical and Experimental Medicine, University of Catania, Catania, Italy

**Keywords:** low urinary tract symptoms, metabolic syndrome, aging, insulin, male PCOS-equivalent, benign prostate hyperplasia

## Abstract

Benign prostate hyperplasia (BPH), one of the most common diseases in older men, adversely affects quality-of-life due to the presence of low urinary tract symptoms (LUTS). Numerous data support the presence of an association between BPH-related LUTS (BPH-LUTS) and metabolic syndrome (MetS). Whether hormonal changes occurring in MetS play a role in the pathogenesis of BPH-LUTS is a debated issue. Therefore, this article aimed to systematically review the impact of hormonal changes that occur during aging on the prostate, including the role of sex hormones, insulin-like growth factor 1, thyroid hormones, and insulin. The possible explanatory mechanisms of the association between BPH-LUTS and MetS are also discussed. In particular, the presence of a male polycystic ovarian syndrome (PCOS)-equivalent may represent a possible hypothesis to support this link. Male PCOS-equivalent has been defined as an endocrine syndrome with a metabolic background, which predisposes to the development of type II diabetes mellitus, cardiovascular diseases, prostate cancer, BPH and prostatitis in old age. Its early identification would help prevent the onset of these long-term complications.

## Introduction

Prostate diseases, usually diagnosed in the elderly, mainly include benign prostatic hyperplasia (BPH) and prostate cancer (PCa). BPH is defined as an increased prostate volume, largely due to the cellular proliferation occurring in the transition zone, namely the portion of the prostatic tissue that surrounds the urethra. BPH is an age-dependent disease which can obstruct the prostatic urethra. Its prevalence varies between 5% and 10% in men aged 40, but reaches 80% in men aged 70–80 (1). PCa represents the second cause of mortality for oncologic diseases in Western countries (2). Although ethnicity also influences the epidemiology of PCa (3), increasing age is a widely-accepted risk factor for the development of this malignant tumor. Accordingly, it is more frequently diagnosed in aging men, whereas it is quite uncommon before the age of 45 (4).

Hormonal changes occurring in aging may play a role in the pathogenesis of prostate diseases. These include changes of sexual hormones, insulin-like growth factor 1 (IGF1), thyroid hormones, cortisol and insulin. Importantly, hyperinsulinemia seems to impact on prostate tissue and is associated with prostate inflammation and hyperplasia. However, the mechanisms of this association are not well-recognized yet. This review aims to provide comprehensive and updated insights into the endocrinology of the aging prostate, including possible pathogenic mechanisms linking prostate to endocrinological and metabolic health.

## Sex Hormones and Prostate in Aging

Prostate diseases and androgen decline represent some of the most common conditions in the aging male. Several large cross-sectional studies have shown an approximate 1%–2% annual decline in testosterone (T) levels in men during aging (5, 6). In middle-age men, the combination of low T and hypogonadism-related symptoms is known as “late-onset hypogonadism (LOH).” LOH has an estimated prevalence of 2.1% (7), but it reaches 15% when only biochemical criteria are used for its diagnosis (8). However, the presence of various diseases plays a crucial role in the decrease of T production during aging. These include obesity, type 2 diabetes mellitus and metabolic syndrome (MetS), which can also lead to an estrogen increase (9). Considering the high prevalence of these diseases in elderly men, it is important to understand the role of sex hormones, and, therefore, of their age-related change, in the development and the treatment for prostate disorders.

### Androgens

#### Androgens and Prostate Physiology

Androgens are essential for the development and the growth of the male genital system, including the prostate. The effects of androgens on the prostate are mainly mediated by the androgen receptor (AR), which is expressed in both prostatic stromal and epithelial cells (10). T, produced by Leydig cells of the testes, is the main circulating androgen in men and it is converted to its more potent metabolite, the 5α-dihydro-testosterone (DHT) by 5α-reductase whose type 2 is the isoform mostly expressed in the prostate tissue (11). Other androgens include adrenally-derived dehydroepiandrosterone (DHEA), androstenedione and 5α-androstenedione, which can be converted to sex steroids with higher potency and act on the prostate.

During development, androgens stimulate differentiation and proliferation of both the epithelial and the stromal compartments of the gland (12). Prostate differentiation and growth start at the 10–12th week of gestation (13). The increase of T that occurs shortly after birth and then, in the period known as “mini-puberty,” has a crucial role in prostate development (13). After mini-puberty, T levels and prostate volume decrease and prostate growth is quiescent until puberty (14). During puberty, a second phase of prostate growth starts and the prostate size increases from about 10 g at early puberty to almost 20 g around the age of twenty (15). In middle age, a third phase of prostate proliferation begins and continues up to the elderly (15). However, this third phase is different from the first two phases of prostate growth. First, the proliferation involves only one of the three anatomical areas of the prostate, the transitional zone surrounding the urethra, while the entire gland grows during the first two phases. Second, an age-related decline in serum T levels begins in middle age. Therefore, although androgens are responsible for prostate growth in the first two phases, the role of androgens is the object of great debate for middle-aged and elderly men (10). Indeed, a clear association between higher circulating T levels and benign prostatic hyperplasia (BPH) has never been demonstrated (16).

#### Androgens and Prostate Diseases

##### Androgens and Benign Prostate Hyperplasia

LUTS and BPH have long been considered a relative contraindication to testosterone replacement therapy (TRT). However, recent studies have contradicted the classic idea that androgens inevitably stimulate prostate growth (17). According to the saturation model, the prostate is sensitive to changes in androgen levels when they occur in a severe low range, but this sensitivity is lost for T levels corresponding to mild hypogonadism or eugonadal. Indeed, prostate ARs become saturated at relatively low T levels and thus, the gland becomes unresponsive to further increases in T levels (18). Experimental and clinical studies suggest that the saturation point for human prostate tissue probably occurs in the very low range of T levels, but a precise cutoff value has not been established; it could vary among men (17). According to this model, the association between T and prostate-specific antigen (PSA) is represented by a sigmoid curve. T levels of 8 nmol/L corresponds to the plateau value below which small changes in T levels cause greater variations in PSA (19). As reported in recent reviews (12, 17), studies evaluating the link between TRT and BPH/LUTS have mostly shown that TRT does not have any effect on BPH symptoms [measured by the International Prostate Symptom Score (IPSS)] and occasionally the IPSS score may decrease (20–22). A recent meta-analysis did not show a worsening of LUTS severity in hypogonadal patients treated with T vs. placebo (23). The safety of TRT in patients with severe LUTS remains to be established and the presence of LUTS of moderate-intensity (IPSS ≤19) is not a contraindication to TRT (23).

However, although current evidence suggests that higher T levels are not involved in BPH development, T seems to have a role in this pathogenic process by stimulating inflammation within prostate tissue. Inflammation has a crucial role in the pathogenesis of BPH and low, rather than high, T may stimulate prostate inflammation (12). Nevertheless, an anti-inflammatory effect of T in the prostate has also been described. In an experimental study using human prostatic stromal cell cultures, the treatment *in-vitro* with different pro-inflammatory stimuli, such as tumor necrosis factor α (TNF-α) or lipopolysaccharide (LPS), or co-culturing with activated CD4β lymphocytes, significantly enhanced the secretion of several cytokines and growth factors (24). The pre-treatment of BPH stromal cells with DHT inhibited the secretion of several inflammatory/growth factors and the proliferation of activated CD4β lymphocytes in a dose-dependent manner (24). The same authors developed a rabbit model of MetS, by feeding New Zealand male rabbits with a high-fat diet (HFD) for 12 weeks (25). Rabbits with MetS developed glucose intolerance, dyslipidemia, hypertension, increased visceral fat accumulation, hypogonadotropic hypogonadism, hyperestrogenism, and prostate inflammation. TRT has been shown to protect rabbits from prostatic hypoxia, fibrosis, and inflammation induced by the high-fat diet that appears to play a role in the development and even progression of BPH/LUTS (25). Interestingly, they found that MetS severity was associated with an increase of *AR* and *estrogen receptor* α (*ERα*), but not of *estrogen receptor β* (ER*β*) gene expression within the prostate (10). This suggests that the prostate could become more sensitive to sex hormone changes that occur during MetS. Indeed, TRT not only corrects the low T levels and the rise of estrogen levels which are typical of MetS, but it also normalizes the majority of MetS-induced prostate alterations (25).

Accordingly, in human BPH stromal cells, AR activation by DHT inhibited TNFα, LPS, or CD4 (+) T cell-induced secretion of inflammatory/growth factors, including interleukin (IL)-6, IL-8, and basic fibroblast growth factor (bFGF), by blocking the nuclear translocation of the nuclear factor kappa-B (NF- κB) (24). Moreover, HFD prostates had increased phosphodiesterase type 5 (PDE5) expression. This was associated with a higher expression of *cyclooxygenase 2* (*COX2)* and *TNFα* among inflammatory genes and of *TGFβ*, *Rho-associated coiled-coil-containing protein kinase 2* (*ROCK2*) and *α spinal muscular atrophy* (*αSMA*) among those genes specifically involved in fibrosis and myofibroblast activation. Interestingly, HFD-induced *PDE5* overexpression was counteracted by T treatment (26). Therefore, recent evidence suggests that the treatment of hypogonadism, which is frequently associated with MetS, not only does not seem to be dangerous for prostate health, but could even prevent the inflammatory process of this gland (12).

Even in inflammation of the prostate caused by *Escherichia coli*, the most frequent bacterial infection due to the reflux of infected urine into the prostate ducts (ascending urethral infection), T seems to play a protective role. Ho and colleagues have shown that T not only inhibits the invasion and colonization of the uropathogenic *Escherichia coli* (UPEC), but has also reduced the levels of pro-inflammatory cytokines (IL-1β, IL-6, and IL- 8) induced by UPEC in a dose-dependent manner. In particular, T plays an anti-inflammatory role in LPS-induced prostate cell inflammation by down-regulating *Janus kinases, signal transducer and activator of transcription proteins* (JAK/STAT1) signaling pathway (JAK/STAT1) (27).

##### Androgens and Prostate Cancer

The concern that T promotes the development of PCa has been the object of great debate since the discovery of PCa dependency on androgen in the 1940s thanks to the pioneering studies of Huggins and Hodges, who found that castration resulted in regression of metastatic PCa (28). However, according to the saturation model, androgen-dependency of prostate growth is evident only in hypogonadism (18). Thus, the relationship between T and the risk of PCa remains poorly understood. Several studies have shown an increased risk of PCa in men with hypogonadism (29). This hypothesis was firstly reported in 1996 by Morgentaler and colleagues, who found a higher prevalence of biopsy-detectable PCa in men with low total or free testosterone levels (30) and it was supported by subsequent studies (31–34). In contrast, studies assessing the relationship between high endogenous T levels and the development of PCa are less clear (29). However, there is evidence that previous T levels may influence Gleason scores, clinical outcomes and recurrence (29).

More controversial remains the safety of TRT in patients with a history of PCa after treatment. T could cause the growth of subclinical cancers that are frequently hidden in the prostate of older men (35). Aside, metastases of PCa are hormone-dependent, therefore T should not be administered to these patients (35). However, some clinicians have suggested considering TRT for patients with a history of organ-confined PCa if they have undergone radical prostatectomy, have undetectable PSA for at least two years and low-grade PCa (Gleason score <7). The possible risks and benefits of TRT should be discussed with the patient and TRT should be followed up with appropriate monitoring by experienced physicians (36).

Also, although adrenal androgens have weak androgenic effects, 11-oxy-androgens of adrenal origin can be metabolized in peripheral tissues to potent androgens which might have a role in the development of PCa. Moreover, emerging evidence suggests the role of microbiome components in the development of PCa that are only beginning to be understood. Recent studies have shown that gut bacteria are capable of metabolizing C21 glucocorticoids to 11-oxygenated C19 androgens *via* an enzyme known as steroid-17,20-desmolase (*desAB*) encoded by the *desAB* gene. A cortisol-inducible operon (*desABCD*) was previously identified in *Clostridium scindens* ATCC 35704 encoding enzyme involved in anaerobic side-chain cleavage. This operon also encodes a transketolase (desAB) which have steroid-17,20-desmolase activity (37). Interestingly, the compounds cleaved by steroid-17,20-desmolase, 11-ketoandrostenedione (11 KT), and 1,4-androstadiene-3,11,17- trione (AT) were found to stimulate proliferation of PCa cells (LNCaP), to a greater extent than dihydrotestosterone. Further researches are needed to fully understand the mechanism through which these compounds stimulate cell proliferation and the potential clinical role in the development and treatment of prostate cancer (38).

### Estrogens and Prostate

Prostate is commonly considered a target of androgens, but also estrogens can play an important role in prostate growth and differentiation. In fact, both ERα and ERβ are expressed in the prostate. ERα is mainly located in the stromal cells and its activation regulates the growth of both stromal and epithelial prostatic cells *via* paracrine mediators like stromal bFGF, epidermal growth factor (EGF), and IGF1 (39, 40). In contrast, ERβ is located in epithelial cells and their activation modulates the estrogenic signals in the prostate (41).

17ß-Estradiol (E_2_) is considered the most potent estrogen in men and it mainly originates from aromatization of T in fat and muscle, whereas about 20% is secreted by Leydig cells (42). In aging men, paralleling the decrease of T levels, the ratio of estrogens to androgens shows an important increase (43). There are several endogenous and exogenous estrogens which may play an important role in prostate. Endogenous estrogens include estrone (E_1_), which is considered to have minimal influence within the prostate, and estriol (E_3_), the main estrogen of pregnancy, which is present in minimal concentrations in men. However, E_2_ can be a potent inducer of prostatic proliferation (44). Local steroids with ER agonist activity include also 5α-androstane-3β, 17β-diol (3βAdiol), and 7α-hydroxy-DHEA (7HD). The effects of these sex steroids are not fully understood but they seem to influence prostate hyperplasia (44). Exogenous estrogens include therapeutic drugs, phytoestrogens, and endocrine disruptors. ERs have a high affinity for environmental estrogens such as bisphenol A (BPA), phthalates, pesticides, etc. In rodent studies, developmental BPA, DES, or E_2_ exposure affects prostate epigenome and thus causes increased prostate susceptibility to dysplasia and hormonal carcinogenesis with aging (45).

Estrogen hormone action in the prostate depends not only on the types of estrogens but most of all, from the type of ER. Specifically, ERα activation is associated with prostate hyperplasia, inflammation, and dysplasia (46). However, the main stimulation of inflammation in BPH by estrogens seem to be mediated by the membrane ER G protein-coupled receptor 30 (GPR30) or G protein-coupled ER (GPER), which are also expressed in prostate stromal cells (47). On the contrary, ERβ inhibits proliferation and its knockout results in prostate hyperplasia (48). The prostatic hyperplasia observed in ERβ knockout mice is attributed to the unopposed action to ERα, suggesting that the ratio ERα/ERβ is an important factor in estrogen-induced proliferation (44).

#### Estrogens and Benign Prostate Hyperplasia

Recent studies suggest that not only low T levels, but also an increase of estrogens may favor BPH/LUTS progression (10). Marmorston and colleagues first reported that the E_2_/T ratio in 24-h urinary collections was elevated in men with BPH compared to normal controls (49). Other epidemiologic studies have found an association between BPH and higher serum estrogen levels or estrogen/androgen ratio (50, 51). As previously reported, prostate inflammation could be amplified and maintained by metabolic alteration occurring in conditions such as MetS. Vignozzi and colleagues showed that HFD rabbits had higher E_2_ to T ratio and lower urinary tract fibrosis, which improved with TRT (25). Recent evidence has also shown that leptin, a hormone produced by adipocytes, induces proliferative effects in prostate cells. This effect may be partially mediated by the direct effect of leptin on estrogen metabolism, as leptin induces aromatase expression (52).

Therefore, ERα, as a key mediator, is also a potential therapeutic target in BPH. The block of conversion of androgens to estrogens by aromatase inhibitors seems to prevent prostate hyperplasia (12). Similar to aromatase inhibitors, selective estrogen receptor modulators (SERMs) have shown anti-proliferative effects on prostate tissue (53).

#### Estrogens and Prostate Cancer

Evidence suggests that ERα mediates the harmful effects of estrogen not only promoting BPH/LUTS, but also prostate carcinogenesis. In the early 1980s, Noble showed the estrogen-dependence of PCa in a rat model (54). In aromatase knockout (KO) mice, high T levels only lead to prostate hypertrophy and hyperplasia, whereas high E_2_ and low T levels induced also premalignant lesions (55). Indeed, prostatic intraepithelial neoplasia does not occur in ERαKO mice and SERMs that bind and inhibit ERα prevent PCa progression in mice and men (56–58). Epidemiological studies have confirmed the role of estrogens on PCa (59). African-American men, who have high serum E_2_ levels, present an increased risk of developing PCa (60). Indeed, ERα expression is significantly associated with high Gleason score and poor survival in PCa patients (61), whereas the expression of ERβ seems decreased or lost in PCa samples (62). Therefore, preclinical and clinical results suggest that ERβ agonists may be useful in PCa therapy, especially in the early stage (59).

Since the discovery of estrogen dependence of PCa, numerous clinical trials were conducted and molecular and functional effects of antiestrogen treatment in PCa have been conducted. Androgen deprivation therapy (ADT) is the first line of treatment for PCa. Nonetheless, ADT frequently induces resistance and PCa can progress toward an androgen-independent form, known as castration-resistant PCa, characterized by worse prognosis. For this reason, alternative approaches to androgen ablation have been investigated to prevent the progression of PCa. Currently, molecular networks of estrogenic signaling *via* ERα, ERβ, and GPR30 in PCa have not been fully understood but new compounds, whose efficacy has been successfully tested in preclinical and clinical models of PC, opened the way for novel therapeutic strategies for treating prostatic diseases (41, 59).

In conclusion, the above-mentioned evidence suggests a role for age-related changes in sex hormones; in particular, hypogonadism and the increase in estrogen levels related to obesity play a pivotal role in the pathogenesis of age-related prostatic diseases.

## Insulin-Like Growth Factor 1 and Prostate

Several studies indicate that IGF1 declines with age (63). Interestingly, IGF1 seems to have an important role in the pathogenesis of metabolic syndrome. Indeed, several studies, both *in-vitro* and *in-vivo* have shown the association between low levels of IGF1 and altered lipid metabolism, cardiovascular disease and diabetes (64). Nevertheless, increased levels of IGF1 have also been reported in patients with diabetes mellitus (65).

A large body of evidence shows that the growth hormone (GH)- IGF1 endocrine axis has a pivotal role in the growth and development of prostate in normal physiology as in pathological conditions (66–69). IGF1 is secreted mainly by the liver under GH stimulation, but it is also expressed locally within the stromal and epithelial cells of the prostate, where it can act in autocrine/paracrine manner (70). The binding of IGF1 to the IGF1 receptor (IGF1R) or the insulin receptor (IR) in the prostate gland, activates the phosphoinositide 3-kinase (PI3K)/protein kinase B (AKT) pathway and RAF/MAPK pathway, which promote cell survival and proliferation (70).

Some studies have shown a greater prevalence of prostate enlargement and BPH in patients with acromegaly (67, 69). Kumar and colleagues documented structural changes in acromegalic patients by prostatic biopsy (71). They found that patients with acromegaly have higher IPSS, an increased rate of prostate enlargement on rectal exploration and ultrasound, higher PSA levels and obstructive pattern on uroflowmetry, and structural prostate changes, regardless of their age, disease activity, or gonadal status (71). These results suggest that the hyper-activation of the GH/IGF1 axis plays an important role in the pathogenesis of BPH in patients with acromegaly. This is further supported by the decrease of prostate volume in patients with inactive disease (for more than 24 months) (71).

Also, there is ample evidence that IGF1 and the signal transduction network that it regulates have important roles in the development of tumors (72). Epidemiological studies have reported that high circulating levels of IGF1 are associated with an increased risk of PCa, particularly advanced disease (70, 73). Experimental studies have shown that the binding of IGF1 to both the IGF1R and IR promotes mitogen signaling events, increases cell proliferation and inhibits apoptosis (72). Both IGF1R and IR are overexpressed in PCa tissue (74). Moreover, the expression of the components of the GH, insulin, and IGF1 axes can be finely modulated in the prostate by environmental factors such as the diet. As previously described, it was well documented that obesity promotes structural changes in the prostate (12). Prostate of obese mice presents altered mRNA expression levels of GH receptor (GHR) and glucose transporter 4 (GLUT4) and an up-regulation in IGF-binding protein 3 (Igfbp3) expression, which might have pathophysiological implications (75). Emerging evidence suggests a direct regulation of IGF/insulin signaling to the *transmembrane serine protease 2* (*TMPRSS2*)*/V-ETS avian erythroblastosis E26 oncogene homolog* (*ERG*) gene fusion, one of the main somatic events in PCa (76). It has been hypothesized that ERG-positive tumors may be more sensitive to IGF/insulin signaling, which could promote PCa progression (70). Furthermore, another possible mechanism is the activation of AR. IGF1 signaling stimulates ARs by attenuating forkhead box-containing protein O subfamily (Foxo1) inhibition (77). Recent researches have highlighted the involvement of not only IGF1 signaling but also of GH itself. GH is responsible for the activation of transcription 5 (STAT5) protein which is involved in the development of several tumors including PCa (78). GHRH antagonists have been shown to decrease directly prostate volume without involving the androgen pathway (79).

Neoplastic, cardiovascular and respiratory disorders are common causes of mortality and morbidity in patients with acromegaly. Nonetheless the higher prevalence of “prostate structural changes” and a possibly increased risk of PCa in patients with acromegaly, no guidelines recommend screening for prostatic disorders. Therefore, a prostatic evaluation may be useful in the work-up of all male patients with acromegaly (71). Actual evidence supports the possible involvement of GH/IGF1 in the pathogenesis of BPH and PCa also in the general population and therapeutic agents targeting the IGF1R may be beneficial in the treatment of prostate diseases.

Finally, the increase of IGF1 seems to play a role in the pathogenesis of BPH and PCa, since it stimulates prostate cell proliferation. Notably, this hormone usually declines with age, although metabolic abnormalities and acromegaly leads to IGF1 increase. Therefore, IGF1 levels should be assessed in patients with prostatic diseases, when diabetes (or other metabolic alterations) or acromegalic features concomitantly occur.

## Thyroid Hormones and Prostate

Thyroid hormones (THs) are involved in cellular growth, metabolism and differentiation. Their effects are mainly mediated by triiodothyronine (T3) that, by binding the nuclear TH receptors (TRs), activates TH response elements (TREs) in the promoter of TH target genes. However, non-classical or non-genomic effects of TH have also been described (80).

The prevalence of thyroid dysfunction increases in the elderly. Recent data from observational studies suggest that serum thyroid-stimulating hormone (TSH) levels increase in older people. The US National Health and Nutritional Examination Survey (NHANES) III study showed that serum TSH concentrations, as well as serum thyroid peroxidase (TPOAb) and thyroglobulin (TgAb) antibodies, increased with age in both men and women (81). Hyperthyroidism is less common than hypothyroidism in the elderly and mainly caused by autonomously functioning thyroid nodules (82).

TRs are strongly expressed in the human prostate (83) and several studies have investigated the role of THs in the development of prostate diseases. Epidemiological studies have found that men with BPH or PCa have significantly increased serum T3 levels compared with euthyroid men (84, 85). Accordingly, Eldhose and colleagues found significantly increased levels of free T3 (FT3) and free thyroxine (FT4) and decreased levels of TSH in patients with BPH compared with controls. They also found that FT3 correlated positively and TSH negatively with prostate volume (86). It has also been shown that patients with hypothyroidism have a decreased risk of developing PCa compared to euthyroid men (87). A recent meta-analysis has shown that hyperthyroidism was associated with higher risks of PCa (pooled risk ratio: 1.35, 95%CI: 1.05-1.74) compared with euthyroidism (88). Experimental studies have shown that THs increase PCa cell proliferation *in-vitro* (89). Specifically, Hsieh and Juang found that T3 can enhance LNCaP cell proliferation and that this effect is cell-specific. LNCaP cells are androgen-sensitive and well-differentiated cells, derived from metastatic lymph nodes of a PCa patient (90). Other studies also indicated that T3 has an important role in the regulation of growth and differentiation of LNCaP cells and PSA expression (91–93). Specifically, a direct effect of T3 on PSA expression was described. Zhu and Young identified a functional TRE in the PSA promoter region, suggesting that T3 regulates PSA expression at the transcription level (93). Also, it has been described that T3 upregulates the proliferation of LNCaP cells through downregulation of the *B-cell translocation gene 2* (*BTG2*), a gene involved in cell-cycle regulation, through the TRE pathway (94). Experimental studies have also shown that THs promote carcinogenesis by inducing angiogenesis (95). Non-classical mechanisms of THs may be also responsible for the effects on the prostate. The binding of TH to plasma membrane receptor integrin avb3 might activate various pro-carcinogenic pathways, including protein inhibitor 3 kinases (PI-3 K) and mitogen activated-protein kinases 1/2 (MAPK 1/2), thereby increasing cell proliferation and angiogenesis (80).

In conclusion, although a relationship between TH and prostate seems clear, the exact mechanisms by which they act need to be further investigated and targeting TH actions might become an alternative adjuvant therapy against PCa proliferation. Due to the age-related rise in TSH levels and the negative correlation between serum TSH levels and prostate volume (81), the role of this hormone in the pathogenesis of prostate diseases in the aging male seems unlikely.

## Role of Insulin in Prostate Diseases

### Insulin, Metabolic Syndrome, and Benign Prostate Hyperplasia

BPH is considered an age-dependent disease, whose etiology is currently poorly understood. Several studies address to insulin-resistance and hyperinsulinemia, which are components of MetS, a role in the pathogenesis of BPH and LUTS. Accordingly, hyperinsulinemia has been shown to enhance prostatic epithelial cell proliferation *in-vitro* (96) and, conversely, hypoinsulinemia decreases prostate volume (97). In line with these findings, patients with serum insulin levels >13 mU/l have a greater prostate volume and annual BPH growth rate compared with those with insulin levels <7 mU/l (98).

Data from the second Nord-Trondelag Health Study, carried out in 21,694 patients, revealed a significantly higher risk for LUTS in patients with diabetes mellitus than in non-diabetic men (99). Also, the risk for developing LUTS has been esteemed twice in patients with diabetes compared with non-diabetic men (100). Accordingly, insulin-resistance is an independent predictor of severe LUTS development (101). This may be attributed to the autonomic nervous system hyperactivity, mainly involving the α-adrenergic pathway, which is closely associated with hyperinsulinemia and is involved in the pathogenesis of LUTS (102).

A recent retrospective study carried out in about 900 patients reported that, after correction for age, insulin levels and insulin-resistance are significantly associated with prostate volume. Interestingly, MetS predicted BPH/LUTS clinical progression (103), thus pointing to the additional role of MetS, other than insulin, in BPH/LUTS. A systematic review with meta-analysis performed on 8,476 participants, including 5,554 (30.1%) with and 12,922 (69.9%) without MetS showed a significantly higher prostate volume in patients compared to controls (104).

The National Cholesterol Education Program adult treatment panel III (2005 revision) defines MetS as the presence of three or more criteria among the following: i) abdominal obesity (waist circumference >102 cm), ii) hypertriglyceridemia (>150 mg/dl) or medications, iii) low high-density lipoprotein (HDL) cholesterol (<40 mg/dl) or medications, iv) hypertension (>130/85 mmHg) or medication, v) high fasting glucose (>110 mg/dl) or medication (105). The evidence supports an association between BPH and each of the MetS components (102).

In greater detail, a BMI >35 Kg/m^2^ has been associated with a 3.5-fold higher risk of developing an increased prostate volume (>40 ml) (106) and with a 1.2-fold higher risk of developing LUTS compared to those with a BMI <25 kg/m^2^ (107). Data obtained in 21,694 patients have confirmed these findings by showing that BMI is a predictor for LUTS development (99). Other reports also support such conclusions (100, 108).

Notably, dyslipidemia is also associated with BPH. Patients with lower HDL cholesterol show increased prostate volume and a higher annual BPH growth rate compared with higher HDL cholesterol values (109). Also, patients with BPH have higher total and low-density lipoprotein (LDL) cholesterol than those without BPH (110) and an association between LDL cholesterol and BPH has been already shown (111). Also, among patients with diabetes, those with the highest LDL cholesterol levels have a 4-fold higher risk of developing BPH (111). These findings suggest that dyslipidemia strongly associates with BPH in the presence of other signs of MetS.

Results from 2,372 patients enrolled in the Third National Health and Nutrition Examination Survey (NHANES III) provide a role for the association between hypertension and LUTS-BPH. Particularly, those with a positive anamnesis for hypertension have increased odds for LUTS compared to those with no hypertension (112). These findings have been largely confirmed also elsewhere (100). Moreover, patients with hypertension showed larger prostate volume and a higher annual BPH growth rate compared with controls (109).

In addition to mechanisms closely associated to hyperinsulinemia, the impairment of nitric oxide (NO) and NO synthase (NOS) activity, the Rho kinase system, the pro-inflammatory status and the abnormality of sexual hormones seems also to be involved in BPH/LUTS pathogenesis in patients with MetS (102, 113). However, the hypothesis of the existence of a male polycystic ovary syndrome (PCOS) equivalent may represent an additional pathogenic mechanism deserving of consideration (please see *Male PCOS Equivalent Exists: A New Syndrome*)*?*.

### Insulin and Prostate Cancer

Several lines of experimental evidence have pointed to the role of insulin in the enhancement of prostate carcinogenesis, particularly in advanced PCa. More in detail, androgens are known to induce epithelial differentiation in prostate cells (114). Androgen deprivation therapy (ADT), usually prescribed in patients with advanced PCa, leads to cellular de-differentiation and trans-differentiation (114–116). Under such conditions, hyperinsulinemia, a common side effect of ADT, enhances PCa cell plasticity, thus increasing tumor migration and invasiveness, by upregulating the Forkhead Box Protein C2 (FOXC2) transcription factor (117). Furthermore, PCa cell lines have an increased ratio of pyruvate dehydrogenase flux to citrate synthase flux following exposure to insulin, which correlates with an insulin dose-dependent increase in cell division (118). This experimental evidence suggests that ADT-induced hyperinsulinemia may likely impact on cancer progression and metastasis.

In line with these *in-vitro* findings, hyperinsulinemia has been associated with a greater PCa mortality (119–122) and treatment failure (122). A retrospective study evaluating the effects of MetS on the time to develop tumor progression in patients with PCa and ADT reported a significantly shorter time to PSA progression (16 vs. 36 months) and a trend toward a shorter overall survival (36.5 vs. 46.7 months) when MetS is present compared with those who do not have it (122). Some Authors have even proposed to define PCa as a component of MetS, since hyperisulinemia prospectively is a risk factor for PCa deaths (119). A long-term survival analysis performed on 2,546 patients who developed PCa, found that patients with weight excess and high C-peptide levels have a four-fold higher risk of mortality compared with those with a BMI <25 kg/m^2^ and low C-peptide levels, independently of other clinical predictors (120).

Supporting these data, the IR is expressed in PCa cells and, interestingly, its expression increases with the Gleason score (74, 123) and ADT (124).

Despite such findings, whether treatment with metformin or other insulin-sensitizing drugs may play a role in PCa therapy is still a matter of debate. An *in-vitro* study reported that GSK1838705A, a potent insulin-like growth factor-1 receptor (IGF1R)/IR inhibitor, is capable of decreasing docetaxel-resistant PCa cell viability and migration, as well as *in-vivo* tumor growth. Therefore, a role for this drug for the treatment of advanced resistant PCa has been postulated (125). Also, by blocking IGF1R, metformin has been reported to significantly inhibit PCa cell proliferation, migration and invasiveness, suggesting a role in PCa treatment (126). Furthermore, tyrphostin NT157, which inhibits the insulin receptor substrates 1 and 2 (IRS1 and IRS2), was shown to decrease proliferation and increase apoptosis of PCa cell lines (127).

On these premises, a population-based study carried out in a cohort of 1,001 patients with PCa and 942 controls showed a lower PCa prevalence in patients using metformin compared with non-users. Also, it showed an inverse relationship between PCa risk and metformin length of treatment, intensity of use and cumulative dosage (128). Similarly, a population-based study on 24,723 case-control pairs found a lower PCa risk in men using antidiabetic medication (129). Other evidence supports a beneficial effect of metformin in lowering PCa incidence and overall survival (130–132).

In conclusion, despite the evidence on the possible role of antidiabetic drugs as adjuvant therapy in patients with PCa, particularly in those on ADT, is growing, there is still insufficient data from randomized trials to suggest their use in the clinical practice (133).

## Male PCOS Equivalent Exists: A New Syndrome?

PCOS is a very common endocrine disorder in women of reproductive age, with a prevalence of 6-15% (134). A genetic background and environmental factors are involved in its etiology. Despite PCOS diagnostic criteria mainly include hyperandrogenism, oligo-ovulation, or anovulation and polycystic ovaries (135), the role of metabolic dysfunction in the pathogenesis of this syndrome is widely accepted. Indeed, up to 75% of patients with PCOS are insulin-resistant and some are hyperinsulinemic (135). Accordingly, the presence of polycystic ovaries, which gave the name to the syndrome, is only one of the many downstream clinical manifestations of PCOS and it is not the pivotal pathogenic factor leading to the development of this syndrome (136, 137).

Since a genetic background has been observed in PCOS, this hereditary predisposition can be potentially inherited by the male sibling of the affected patients. Interestingly, the brothers and the relatives of women with PCOS have a high prevalence of hormonal and metabolic abnormalities (138–140). They also show a greater prevalence of early-onset androgenetic alopecia (AGA) (141), which has been suggested as a clinical sign of the male PCOS equivalent (142–144). The occurrence of hormonal and metabolic abnormalities in men with early-onset AGA (younger than 35 years) has been reported (145, 146) (**Table 1**). A meta-analytic study performed in 1009 unrelated men found increased luteotropic hormone (LH) and dehydroepiandrosterone (DHEAS), decreased sex hormone-binding globulin (SHBG), a downward trend for FSH and an upward trend for the LH/FSH ratio in patients with early-onset AGA compared with controls. This hormonal pattern somewhat resembles that found in female PCOS. The same meta-analysis showed a significant increase in insulin levels and HOMA index, total and LDL cholesterol and triglycerides in patients vs. controls, already before the age of 35 (147).

**Table 1:** Biochemical and clinical parameters in male relatives of women with polycystic ovarian syndrome, in young and elderly men with early androgenetic alopecia (146).

Parameter	Male relatives of women with PCOS	Men with early-onset androgenetic alopecia	Clinical findings in elderly men with AGA
Serum free testosterone levels	–	↑	–
Serum SHBG levels	–	↓	–
Free testosterone index	–	↑	–
Serum LH levels	–	↑	–
Serum FSH levels	–	↓	–
LH/FSH ratio	–	↑	–
LH and FSH response to GnRH analog	↑	–	–
Serum AMH levels	↑	–	–
Serum DHEAS levels	↑	↑	–
Serum 17α hydroxy-progesterone	–	↑	–
Serum adiponectin levels	↑	–	–
Serum glucose levels	–	↑	–
Serum insulin levels	↑	↑	↑
Risk for insulin-resistance	↑	↑	–
Serum cholesterol levels	↑	↑	–
Risk for metabolic syndrome	–	↑	↑
Risk for type II diabetes mellitus	–	–	↑
Risk for endothelial dysfunction	↑	–	–
Blood pressure	↑	↑	↑
Serum aldosterone levels	–	–	↑
Serum fibrinogen levels	–	–	↑
Risk for atheromatous plaques	–	–	↑
Risk for ischemic heart disease	–	–	↑
Risk for benign prostate hyperplasia	–	–	↑
Risk for prostate cancer	–	–	↑

↑, increased; ↓, decreased; -, non-reported.

AGA, androgenetic alopecia; AMH, anti-Müllerian hormone; DHEAS, dehydroepiandrosterone sulfate; FSH, follicle-stimulating hormone; GnRH, gonadotropin-realizing hormone; LH, luteinizing hormone; SHBG, sex hormone binding globulin.

Large cohort studies in older men with early-onset AGA have clearly shown a higher prevalence of type II diabetes mellitus (148) and a greater risk for cardiovascular disease (CVD) (149). Thus, AGA has been suggested as an independent predictor of mortality for diabetes mellitus and CVD (150). Curiously, these men have also a higher prevalence of prostate diseases, such as BPH (151–155), PCa (156), and prostatitis (157), which are known to be influenced by the metabolic status. In this view, prostate diseases may be seen as long-term complications of the male PCOS equivalent syndrome (**Table 2**) (146). This concept supports the association between metabolic abnormalities and prostate diseases in the elderly. The acknowledgment and timely diagnosis of this syndrome may be of great utility to early identify and treat metabolic disorders in men, preventing the long-term complications, including diabetes, CVD and prostate diseases.

**Table 2:** Age-related features of the male polycystic ovarian syndrome (PCOS) equivalent.

Age	
**<35 years**	Clinical signs of hyperandrogenism (early-onset AGA, acne or hypertrichosis)
	PCOS-like hormonal pattern (e.g., increased DHEAS, 17α-OH-progesterone, FAI, LH/FSH, decreased FSH)
	Metabolic abnormalities (insulin-resistance, low SHBG levels, hyperglycemia, hyperinsulinemia), and/or a trend towards higher BMI values
	A familiar history positive for PCOS
**Elderly men**	Diabetes mellitus, cardiovascular diseases, benign prostatic hyperplasia, prostatitis, prostate cancer

## Conclusion

**Figure 1:**
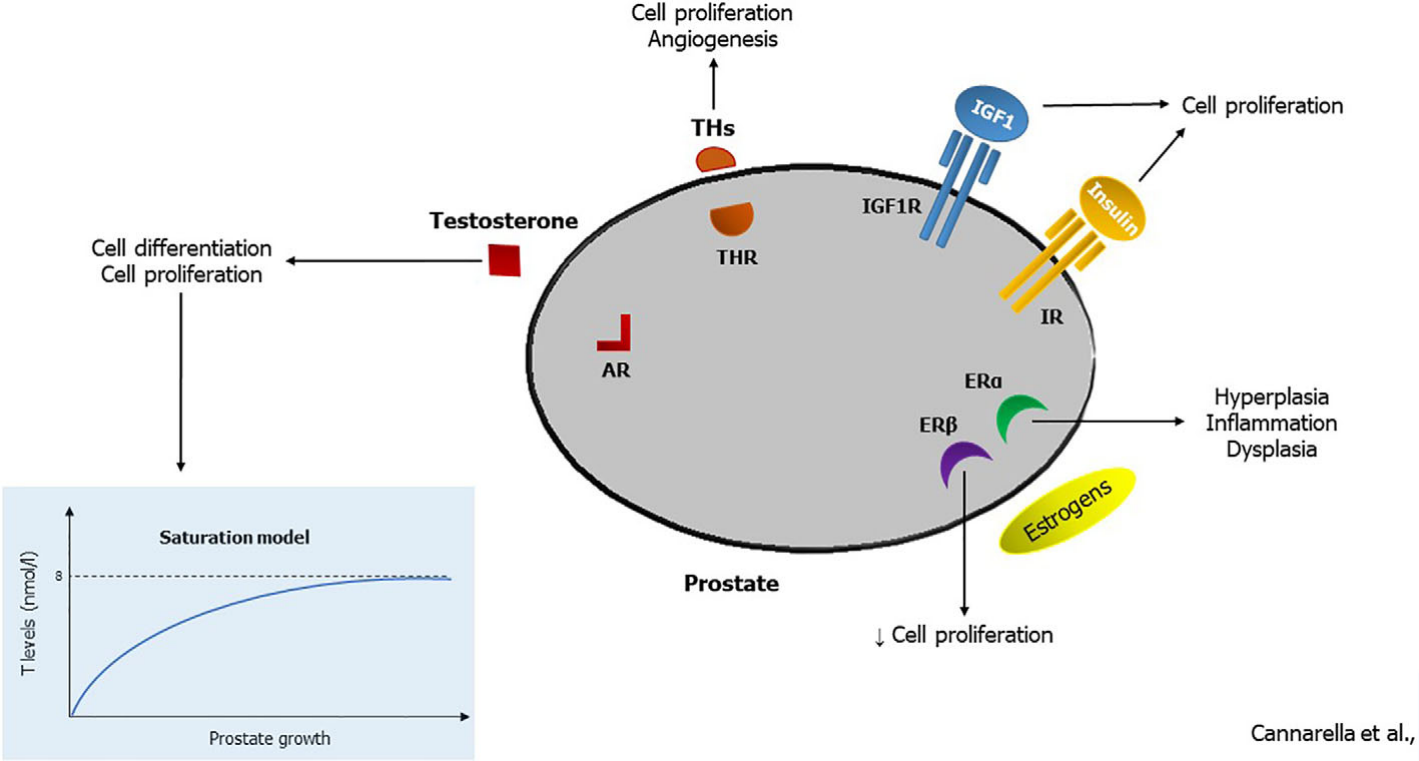
Hormonal effects in the prostatic tissue. By binding to the androgen receptor (AR), testosterone (T) stimulates cell differentiation in prenatal life. According to the saturation model, it induces cell proliferation when its serum levels are in the hypogonadal range (<8 nmol/l). Thyroid hormones (THs) enhance prostate cell proliferation and angiogenesis, triggering the TH receptor (THR). Insulin-like growth factor 1 (IGF1) and insulin induce cell proliferation by interacting with their receptors (IGF1R and IR, respectively). Finally, estrogens can trigger the estrogen receptor α (ERα) that stimulates cell hyperplasia, inflammation, and dysplasia, or the estrogen receptor β (ERβ) that hinders cell proliferation.

In conclusion, the receptors for sex hormones, IGF1, THs and insulin expressed in the prostate cells indicate the close relationship between the prostate and hormones (**Figure 1**). Also, serum levels of these hormones vary with aging and are influenced by several comorbidities such as gonadal dysfunction, thyroid disease, obesity, MetS, insulin-resistance and diabetes mellitus. In particular, the evidence attributes a role in the pathogenesis of prostate inflammation, BPH and PCa to the age-related change in T levels as well as the increase in obesity-related estrogen levels. Furthermore, the increase in IGF1, which occurs in diabetic or acromegalic patients, leads to the proliferation of prostate cells. In contrast, the age-related decline in serum TSH levels is unlikely to be involved in the pathogenesis of prostate disease. Finally, metabolic abnormalities can lead to insulin (but also to IGF1 and E_2_)-mediated prostate inflammation and hyperplasia. This justifies the importance of endocrine counseling in patients with prostate diseases, which primarily allows the identification of endocrine or metabolic comorbidities responsible for an increased cardiovascular risk. Further research is needed to confirm the existence of a male-PCOS equivalent.

## Author Contributions

RC conceived the study and contributed to writing the draft of the paper. RAC contributed to write the draft of the paper and critically revised the final version of the paper. FB searched the studies and contributed to write the draft of the paper. SL contributed to writing the final version of the paper and critically revised it. AC was the project administrator. He contributed to writing the final version of the paper and critically revised it. All authors contributed to the article and approved the submitted version.

## Conflict of Interest

The authors declare that the research was conducted in the absence of any commercial or financial relationships that could be construed as a potential conflict of interest.
